# B’more Healthy Corner Stores for Moms and Kids: Identifying Optimal Behavioral Economic Strategies to Increase WIC Redemptions in Small Urban Corner Stores

**DOI:** 10.3390/ijerph16010064

**Published:** 2018-12-27

**Authors:** Caroline R. Wensel, Angela C.B. Trude, Lisa Poirier, Riyad Alghamdi, Antonio Trujillo, Elizabeth Anderson Steeves, David Paige, Joel Gittelsohn

**Affiliations:** 1 Human Nutrition Program, Department of International Health, Johns Hopkins Bloomberg School of Public Health, 615 N. Wolfe St, Baltimore, MD 21205, USA; atrude1@jhu.edu (A.C.B.T.); lpoirie4@jhmi.edu (L.P.); riyad.q.a@gmail.com (R.A.); jgittel1@jhu.edu (J.G.); 2 Health Systems Program, Department of International Health, Johns Hopkins Bloomberg School of Public Health, 615 N. Wolfe St, Baltimore, MD 21205, USA; atrujil1@jhu.edu; 3 Department of Nutrition, University of Tennessee, Knoxville, Tennessee, 1215 W. Cumberland Ave., Knoxville, TN 37996, USA; eander24@utk.edu; 4 Department of Population, Family and Reproductive Health, Johns Hopkins Bloomberg School of Public Health, 615 N. Wolfe St, Baltimore, MD 21205, USA; dpaige@jhu.edu

**Keywords:** nutrition intervention, public health, food environment, underserved populations, behavioral economics, WIC

## Abstract

Special Supplemental Nutrition Program for Women, Infants, and Children (WIC) redemption rates have been declining in many low-income urban settings, potentially related to aspects of the food environment. B’more Healthy Corner Stores for Moms and Kids was a feasibility trial in Baltimore City that aimed to test multiple behavioral economic (BE) strategies in 10 corner stores (intervention = eight stores, comparison = two stores), to evaluate their influence on the stocking and redemptions of WIC foods. Tested strategies included in-person storeowner training, point of purchase promotion, product placement, and grouping of products in a display. All four strategies were feasible and implemented with high reach, dose delivered, and fidelity. Additionally, text messaging was found to be an acceptable form of intervention reinforcement for storeowners. Analyses to assess change in stocking of WIC foods, total sales of WIC foods, and sales of WIC foods to WIC clients, revealed consistent positive changes after implementation of the store owner training strategy, while changes after the implementation of other strategies were mixed. Furthermore, WIC food sales to WIC clients significantly increased after the simultaneous implementation of two strategies, compared to one (*p* > 0.05). Results suggest that store owner training was the most influential strategy and that the implementation of more BE strategies does not necessarily lead to proportional increases in stocking and sales. Selected BE strategies appear to be an effective way of increasing stocking and sales of WIC foods in small urban food stores.

## 1. Introduction

Poor diet quality is one of the largest risk factors for death and morbidity in the United States and is related to healthy food availability, food insecurity, and obesity [1,2]. In Baltimore City, 23.5% of residents live in healthy food priority areas (previously referred to as food deserts) where small corner stores are a convenient, frequent food source [3,4], and nutrient poor, calorie dense food is highly available [5]. Additionally, 73% of food insecure adults living in areas that lack access to healthy foods in Baltimore City are also obese [6]. 

Revisions to governmental assistance nutrition program guidelines to address dietary requirements and obesity, such as those by the Special Supplemental Nutrition Program for Women, Infants, and Children (WIC), have improved the food environment by increasing access to healthy foods and beverages [7,8]. However, the most current data available indicate declines in WIC client participation. From 2013–2014, there was a 4.6% decline in WIC client participation in Baltimore City compared to only a 1.9% decline in the state of Maryland [9]. As 33% of corner stores in Baltimore City participate in the WIC program and 30% of low-income Black Americans report using WIC vouchers in small food stores [10], challenges specific to WIC implementation in corner stores may deter clients from taking full advantage of the WIC program and thus be partially responsible for declines in participation. In corner stores, barriers to WIC redemption—shared by corner store owners and customers—include perceived lack of demand for WIC-eligible foods, difficulty promoting WIC-eligible foods, difficulty stocking perishable foods, language and cultural barriers, embarrassment at checkout, and difficulty locating products in-store [10,11]. Results from observational and qualitative studies assessing barriers to WIC redemption, although limited, have suggested that the use of behavioral economic (BE) strategies may be an effective means for improving WIC redemption and participation [12]. 

Behavioral economics is widely recognized as having the potential to achieve immense public health impact at low-cost [13]. BE principles, such as nudging—defined as setting defaults, framing, or adding decoy options to alter an individual’s behavior in a predictable manner without forbidding any options or significantly changing economic incentives [14,15]—have proved to be successful at modifying behavior in a variety of preventive health domains [13], including consumer food choice [16,17,18]. Food choice is often subject to temporal factors such as convenience; and, BE techniques such as defaults and framing inherently play to an individual’s impulsive desire to respond to immediate stimuli [19]. In supermarkets, successful applications of BE principles for the modification of consumer food behavior have included grocery cart placards, point of purchase promotions (POP), arrows on store floors, and shelf space management [16,20,21]. And, changes to school lunch programs, such as making the healthier choice the default or easier choice, have successfully modified children’s food consumption [22,23,24]. 

Previous intervention trials in Baltimore City have utilized BE techniques, such as POP posters and shelf and menu labels, in small food stores and carryout restaurants to promote WIC-eligible foods and modify consumer food behavior, with mixed results [25,26,27]. The Baltimore Healthy Eating Zones intervention showed improvements in youth food knowledge but did not impact purchasing behavior [27]; whereas, the recently completed B’more Healthy Communities for Kids intervention trial found significant positive impacts on youth healthy food purchasing behavior but not overall dietary quality [25]. However, research reviewed by the U.S. Department of Agriculture Economic Research Service suggests that specific cues (e.g., appearance, name, price, and information) have the propensity to influence WIC client food choices and thus dietary quality [28]. 

Little research has tested and compared the implementation and impact of various BE strategies. And, no studies have attempted to identify optimal BE strategies for shifting the stocking and sales of WIC-eligible foods by implementing and comparing multiple BE strategies in small food stores. Thus, the objective of the present study was to test multiple BE strategies, alone and in combination, in small urban corner stores, in order to evaluate its influence on stocking and sales of WIC foods. The specific aims were:

(1) To evaluate change in stocking and sales of WIC foods in small urban corner stores by four different BE strategies implemented separately (Treatment 1).
(2) To evaluate change in stocking and sales of WIC foods by combined BE strategies (Treatments 2–4).
(3) To evaluate change in consumer selection and purchasing of WIC foods by four different BE strategies, implemented either alone or combined (Treatments 1–4).

## 2. Materials and Methods 

B’more Healthy Corner Stores 4 Moms and Kids (BHMK) was a randomized, controlled feasibility trial in 10 corner stores (intervention = eight, comparison = two) seeking to identify optimal low-cost BE strategies to increase WIC participation in Baltimore City, MD, USA. 

### 2.1. Setting Description

The Baltimore City food environment consists of 633 corner stores, 185 convenience stores, 47 supermarkets, and six public markets [4]. Approximately 63% of Baltimore City residents are Black and 21.8% of residents live below the poverty line [29]. Additionally, Black residents are most likely to live in a healthy food priority area; 31.5% of Black residents compared to 8.9% of White residents live in healthy food priority areas [4]. 

### 2.2. Overview of Intervention

BHMK was a one-year funded pilot intervention. Recruitment and baseline data collection occurred from February through March 2017; intervention implementation and post-intervention data collection was conducted from April 2017 to January 2018. The trial employed the BE principle of nudging and was informed by social cognitive theory [30,31,32,33,34] and the social ecological model [35]. In accordance with these theoretical frameworks, BHMK attempted to modify food behavior and create environmental level changes by implementing four low-cost BE strategies alone and in combination, working with corner store owners to increase WIC redemption by individual WIC clients. Social marketing strategies (i.e., POP posters and shelf labels) were used to improve knowledge, self-efficacy, and healthy eating intentions of WIC clients and generate demand for healthier foods at corner stores. Additionally, as individuals and the environment have a reciprocal relationship [35], BHMK worked with store owners to implement environmental changes (i.e., product placement and grouping of products in a display) to make healthy food the easy choice for the broader community. 

### 2.3. Corner Store Recruitment and Sample

Participating stores were recruited in low-income healthy food priority areas in Baltimore City, MD, and included ten corner stores. The BHMK study team obtained a list of WIC-authorized stores (*n* = 103) [36] from the Johns Hopkins Bloomberg School of Public Health Center for a Livable Future [37] and screened stores based on the following eligibility criteria: (1) currently WIC-authorized, (2) willing to participate, (3) willing to share stocking and sales data, (4) located in a healthy food priority area. The study team visited 96 corner stores. Twelve of 96 WIC-authorized stores (12.5%) visited by the study team, both met eligibility criteria and agreed to participate in the study. Reasons for non-participation included loss of WIC authorization, recent change in store ownership, and an explicit desire not to participate. Of the 12 stores selected for participation, two dropped out of the study during the implementation of the first BE treatment, reporting the intervention disruptive to normal business, and resulting in a completion rate of 83% (10 stores). Participating stores had similar Healthy Food Availability Index (HFAI) scores to other WIC-authorized stores that did not participate in the program [36], and were located in North, South, East and West Baltimore City healthy food priority areas. There was at least a half mile radius between stores, except two stores that were located less than a quarter mile from each other but separated by a busy road. Additionally, one store had previously participated in a nutrition intervention program, although non-WIC related and all promotional materials were removed prior the commencement of the BHMK intervention. Participating corner store owners self-identified as the following ethnicities 8.3% Black, 16.7% Caucasian, and 75.0% Asian. Of the corner store owners who identified as Asian, 11.1% identified as Indian, 22.2% identified as Chinese Mandarin, and 66.7% identified as Korean American. 

### 2.4. BHMK Intervention

Following recruitment and baseline data collection, participating stores were randomized to one of five treatments (Table 1), where two stores received each treatment type. Store visits were conducted on weekdays between 10:00 AM and 3:30 PM in teams of two to three interventionists and data collectors. Treatment 1 involved the delivery of only one BE strategy. Starting with Treatment 2, additional BE strategies were added one by one with each treatment. BE strategies implemented included: store owner training, point of purchase (POP) promotion, product placement, and grouping of products in a display (Table 1). Strategies did not differ in successive treatments.

#### 2.4.1. Store Owner Training 

Two videos were developed and available in three languages (Korean, Chinese/Mandarin, and English). Video one, 4:16 minutes in length, discussed the benefits of maintaining WIC eligibility and reviewed the stocking of WIC-eligible products and challenges that corner store owners may face. Video two, 9:48 minutes in length, discussed strategies for corner store owners to increase their WIC product sales and introduced corner store owners to forms of verbal encouragement or nudging techniques. During week one of implementation, corner store owners, with oversight of project interventionists, viewed the two videos on an iPad in their preferred language. During weeks two though four of implementation, interventionists reviewed a nudging guide with each corner store owner. The nudge guide consisted of four reminders/ nudges for corner store owners on ways to help their WIC customers (e.g., “Walk your customers through your store to show them where WIC products are located”) and provided sample phrasing (e.g., “Look for foods with WIC labels”). Following each week’s training corner store owners answered four knowledge questions to assess information retention.

#### 2.4.2. Point of Purchase Promotion 

Posters and shelf labels were developed by study and Maryland WIC staff. A total of four posters were developed and differed in color and promoted foods for four WIC-eligible developmental age groups (Supplemental Figure S1). Poster colors were chosen based on previous use in the state of Maryland WIC materials [38]. Shelf labels were developed for each WIC-eligible food and were store specific. Additionally, shelf labels were marked by colored (i.e., pink, orange, blue, green) thumb prints to match the age group posters (Supplemental Figure S2). The purpose of the thumb prints was to help WIC clients, regardless of language or reading level, quickly and efficiently identify eligible age group foods. Posters and shelf labels were hung in all intervention stores during the POP promotion treatment in high visibility areas with approval of the corner store owner (Supplemental Figure S3). Posters and shelf labels were maintained by the project interventionists during each POP promotion treatment.

#### 2.4.3. Product Placement 

In cooperation with corner store owners, the project interventionists moved WIC products to either the front of the store or to eye level; and in some instances, WIC products were moved to both the front of the store and eye level. 

#### 2.4.4. Grouping of Products in a Display 

Corner store owners were offered six possible display racks and asked to choose one. Choices ranged in size from 18 to 72 inches and were made of various materials, including wicker baskets and restaurant quality steel. Each chosen display was ordered, delivered, and assembled in each corner store in a location decided in cooperation between the project interventionist and the respective corner store owner.

#### 2.4.5. Text Messaging Component 

All eight intervention corner store owners were enrolled in a text messaging service (i.e., Mobile ViP, EZ texting) at the beginning of the intervention. Text messages reinforced the BE strategy or combination of BE strategies employed in each store at that time. Similar to the training materials, text messages were available in Korean, Chinese/Mandarin and English. Corner store owners received two to three text messages (e.g., “Do you know how to keep WIC in your store? 1. Keep your store clean! 2. Stock WIC items and fresh foods 3. Make sure the WIC sign is posted!”) per week during each intervention treatment. Text messages were only sent during treatment periods. The text messaging component was assessed in terms of acceptability which was defined as the willingness of store owners to accept the text messaging intervention strategy by remaining enrolled in the text messaging service for the duration of the intervention. Similar definitions of acceptability have been previously used [26]. 

### 2.5. Data Collection and Instruments 

Prior to the start of the BHMK intervention, a baseline Store Impact Questionnaire (SIQ) was completed to assess corner store owner characteristics, including decisions regarding food stocking and self-efficacy to stocking healthier products. The SIQ was repeated post-intervention. At pre- and post-intervention and at each intervention treatment (83 times total), BHMK data collectors conducted an Environmental Assessment (EA), which assessed stocking and owner-reported sales of WIC foods in the prior 7 days, and total WIC redemption in the prior 30 days. Store owners were compensated for their time, after the completion of each EA and SIQ, with a ten-dollar gift card. 

### 2.6. Process Evaluation 

Process evaluation, defined in terms of reach, dose delivered, and fidelity, was conducted during each week of the intervention to monitor implementation quality. Implementation standards were set *a priori (*Table 2) based on standards utilized in similar study populations and settings [39,40,41,42]. Johns Hopkins University undergraduate and graduate students, as well as study staff were trained and performed all data collection and intervention activities. 

### 2.7. Data Management 

Data managers worked with data collectors to ensure all forms were complete without missing or ambiguous entries. Data was then entered and cleaned by the data manager and research assistants into Microsoft Access^®^ (Microsoft Corporation, Redmond, WA, USA, 2016).

### 2.8. Data Analysis

Prior to and for the purposes of analysis, WIC foods were divided into seven groups based on the 2017 Maryland WIC-Authorized Foods List (Supplemental Table S1). Descriptive analyses were conducted to assess changes in stocking of WIC foods, sales of WIC foods to all customers, and sales of WIC foods to WIC clients, after implementation of one BE strategy (Treatment 1). Observed stocking and reported unit-sales were used to calculate changes before and after each intervention implementation. Bivariate linear regressions were used to assess differences in stocking and sales from baseline to post implementation of two, three, and four BE strategies compared to one BE strategy, regardless of the type of the strategies. Our model specification checks, including assessment of model residuals, revealed that treating stocking and sales in it’s continuous shape did not violate linearity assumptions. Regressions were clustered at the store-level to allow for within group correlation. BE strategies were treated as ordinal variables. Statistical significance was set at *p* < 0.05. Statistical analyses were conducted in STATA 14 (Stata Corp., College Station, TX, USA).

### 2.9. Institutional Review Board Approvals 

The BHMK intervention trial was Institutional Review Board (IRB) exempt, as it was not considered as human subjects research by the Johns Hopkins University IRB. 

## 3. Results

### 3.1. Implementaion

We found that it was possible to implement the four BE strategies—store owner training, POP promotion, product placement, and grouping of products in a display—with high reach, dose delivered, and fidelity based on *a priori* set standards for process evaluation (Table 2). Reach and dose delivered were each implemented at 100% or higher for all four BE strategies. Fidelity, was 100% or higher for store owner training, POP promotion, and grouping of products in a display, and above 78% for product placement. With respect to the text-messaging component, 100% of text messages were marked as delivered and 100% of corner store owners remained in the text messaging service for the duration of the intervention. 

### 3.2. Changes in Stocking and Sales Following Implementation of One BE Strategy, All Food Groups Combined

We first combined the seven WIC food groups (Supplemental Table S1) and assessed changes in the three outcomes—stocking of WIC-eligible foods, total sales of WIC-eligible foods to all customers, and sales of WIC-eligible foods to WIC clients—after implementation of one BE strategy, Treatment 1 (Table 3). No statistically significant changes were observed. Store owner training was the only BE strategy with positive trends, although not significant, in all three outcomes. 

### 3.3. Qualitative Changes in Stocking and Unit Sales Following Implementation of One BE Strategy, by Food Group

Changes in the sales and stocking of WIC foods, following the implementation of one BE strategy, were qualitatively assessed in the seven WIC food groups (Table 4). We chose to present a qualitative analysis of observed changes by food group as our sample size was small and regression models yielded statistical noise. Store owner training yielded the most consistent positive trends for each of the three outcomes and seven WIC food groups; whereas, changes following the implementation of other BE strategies showed mixed directionality and varied by outcome and food group.

With respect to changes in the stocking of WIC foods, a consistent positive trend was observed across all four strategies for the infant food group. Positive trends were also observed after the implementation of store owner training, POP, and product placement for the protein and cereal food groups. 

Changes in total unit sales of WIC foods to all customers following store owner training yielded a positive trend for all seven food groups. Changes in the unit sales of WIC foods to WIC clients following store owner training were positive for all food groups except sales of infant foods (Table 4). And, following the implementation of all BE strategies except the grouping of products in a display, sales of dairy to WIC clients trended positive (Table 4). 

### 3.4. Changes in Stocking and Unit Sales Following Implementation of Two, Three, and Four BE Strategies

For all food groups combined, changes in the three primary outcomes—stocking of WIC-eligible foods, total sales of WIC-eligible foods to all customers, and sales of WIC-eligible foods to WIC clients—following the implementation of two, three, and four BE strategies compared to one BE strategy were not statistically significant (Table 5). However, a positive trend was observed following the implementation of two BE strategies. 

We next assessed changes for each of the seven food groups following implementation of two, three, and four BE strategies (Supplemental Table S2). There was a significant increase in the number of WIC-eligible infant foods sold to WIC clients following the implementation of two BE strategies (β = 45.9; 95% CI: 16.6, 75.1; *p* < 0.05), and a significant decrease in the average number of WIC-eligible infant foods stocked following the implementation of three BE strategies (β = −78.4; 95% CI: −150.7, −6.1; *p* < 0.05). No other statistically significant changes in stocking or unit sales were observed for individual food groups (Supplemental Table S2). 

Consistent positive trends in stocking following the implementation of two, three, and four BE strategies were observed in the dairy food group, the grains foods group, and the juice food group; while consistent negative trends in stocking were observed in the infant food group, protein food group, and cereal food group. Consistent positive trends in total unit sales of WIC foods to all customers, following the implementation of two, three, and four BE strategies were observed in the infant food group and the juice group; while, consistent negative trends in total unit sales were observed in the fruit and vegetables group. Consistent positive trends in unit sales of WIC foods to WIC clients, following the implementation of two, three, and four BE strategies, were observed in the infant food group, fruit and vegetable group, and the grains group; there were no consistent negative trends in unit sales of WIC foods to WIC clients observed (Supplemental Table S2). 

## 4. Discussion

To our knowledge, this is the first study to report on the implementation and impact of multiple behavioral economic (BE) strategies on the stocking and sales of WIC-eligible foods in small urban food stores. Results from the BHMK intervention trial demonstrate that it is feasible to implement four BE strategies (i.e. store owner training, point of purchase promotion (POP), product placement, and grouping of products in a display) both alone and in combination in WIC-authorized corner stores in Baltimore City, MD with high reach, dose delivered, and fidelity. Additionally, results suggest that text messaging was an acceptable form of reinforcement, given that all store owners remained in the program.

Store owner training appeared to be the most consistently influential form of BE intervention strategy, whereas results following implementation of other strategies were mixed. Our findings suggest that more BE strategies do not necessarily lead to increased sales and stocking of these foods. We postulate that store owner training had the most influence on stocking and sales of WIC foods because of the intensity of implementation. Store owners were required to watch the same training videos and answer knowledge questions repeatedly. Once learned, knowledge could not be removed. Whereas strategies like POP or grouping of products in a display, which have been previously demonstrated to impact food behavior [25], required physical items (e.g., posters, shelf labels, display racks) which were easily damaged or removed. Several investigators have demonstrated the importance of training as an approach for improving the stocking and sales of healthier products in small stores [26,43]. 

Research suggests that subtle cues such as product placement should be effective at reducing consumption of unhealthy foods and increasing the purchase and consumption of healthier foods [28]; however, this was not observed in the small urban corner stores that participated in our study. During product placement implementation, corner store owners relayed to project interventionists that they and their customers had difficulty finding WIC products in the new locations (i.e., eye-level and/or front of store). While a BE strategy such as product placement may be helpful in a larger grocery store where stock is routinely moved and customers are unfamiliar with the organization of the store, corner store stock is often limited and not routinely moved [10]. Furthermore, it was observed that WIC clients who regularly redeem their benefits in the same corner stores know and have something akin to a familial relationship with corner store owners. This contrasts previous research which suggests extensive challenges between corner store owners and customers [10,11]. It is noteworthy that corner store owners who agreed to participate in the BHMK study may have been particularly invested in the health and well-being of their customers and surrounding community, and also had a better-than-average relationship with their WIC clients. 

The study had several limitations. General declines in WIC sales associated with electronic WIC (eWIC) introduction may have impacted findings. eWIC implementation in Baltimore City commenced 1 July 2017, around the same time BHMK Treatment 2 (implementation of two BE strategies) was completed. After Treatment 2, we observed declines in total unit sales of WIC-eligible foods to all customers (Supplemental Table S2) and average WIC redemption (Supplemental Figure S4). During eWIC implementation corner store owners received training from Maryland WIC on new implementation procedures and the use of EBT machines [44], however project interventionists observed numerous challenges including WIC clients forgetting their EBT pin numbers, EBT machines malfunctioning, and corner store owners having difficulty programing store food prices into the EBT machine. These observations seem to contradict findings in a previous study reviewing the impact of the transition to EBT in WIC, specifically that EBT reduced stigma and embarrassment [45]. Other challenges included observed changes in customer purchasing behavior during intervention implementation. Prior to the implementation of eWIC, a WIC client was required to redeem every item listed on her check during one store visit. However, eWIC permitted WIC clients to split up purchases. Corner store owners anecdotally reported to study interventionists that WIC clients were making more frequent, but smaller purchases. 

Other limitations include the small sample size and sales being a corner store owner self-reported measure. Lastly, there may have been the potential for selection bias, as the 12.5% of the store owners who agreed to participate in our study may differ from overall owners of WIC-authorized stores. Thus, results of this study have limited generalizability. 

Recommendations for future trials include (1) increasing the study duration and sample size to account for seasonality and baseline variability; (2) using and reporting process evaluation measures to monitor intervention implementation and provide context for intervention results; (3) refining and testing optimal store owner nudging and training strategies.

## 5. Conclusions

The literature describes various ways BE strategies have been used to influence food choice and purchasing behaviors but lack a comparison of optimal strategies. This study provides a first attempt at making these comparisons and serves as a reference for future interventions aiming to modify food behavior though BE strategies. In addition, while the use of BE strategies to influence food behavior is increasingly common, no studies have compared the implementation and impact of multiple BE strategies for government food assistance programs. This was one of the first intervention trials to do so and therefore the data provided will be helpful for developing sustainable interventions employing BE strategies, and supporting policies.

## Figures and Tables

**Table 1:** B’more Healthy Corner Stores for Moms and Kids study design and timeline.

Time	Stores 1–2	Stores 3–4	Stores 5–6	Stores 7–8	Stores 9–10
Months 1–2	Recruitment, Baseline Data Collection, Material Development				
Month 3, Treatment 1	Store Owner Training	POP Promotion	Product Placement	Grouping of Products	Control
Month 4	No Treatment				
Month 5, Treatment 2	Store Owner Training, POP Promotion	Store Owner Training, POP Promotion	Store Owner Training, Product Placement	Store Owner Training, Grouping of Products	Control
Month 6	No Treatment				
Month 7, Treatment 3	Store Owner Training, POP Promotion, Product Placement	Store Owner Training, POP Promotion, Product Placement	Store Owner Training, Product Placement, Grouping of Products	Store Owner Training, Product Placement, Grouping of Products	Control
Month 8	No Treatment				
Month 9, Treatment 4	Store Owner Training, POP Promotion, Product Placement, Grouping of Products	Store Owner Training, POP Promotion, Product Placement, Grouping of Products	Store Owner Training, POP Promotion, Product Placement, Grouping of Products	Store Owner Training, POP Promotion, Product Placement, Grouping of Products	Control

Abbreviations: POP (point of purchase); Treatment 1 (one behavioral economic strategy), Treatment 2 (one behavioral economic strategy paired with store owner training), Treatment 3 (store owner training plus two behavioral economic strategies), Treatment 4 (all four behavioral economic strategies).

**Table 2:** Process evaluation of behavioral economic strategy implementation in the BHMK program: Percent of High Standard Met.

Process Standard		High Standard Set	Percent of High Process Evaluation Standard Met ^a^				
			T1 ^b^	T2 ^b^	T3 ^b^	T4 ^b^	Overall ^c^
No. of stores participating in BHMK program throughout intervention		≥8	100	100	100	100	100
No. of times BHMK team meets with each store owner per week		≥1	100	100	100	100	100
Store Owner Training							
	No. of (in-person) training videos watched by each corner store owner per treatment ^d^	2	100	100	100	100	100
	Percentage of correct training video questions	≥75%	110	123.3	123.5	133	122.5
Point of Purchase Promotion							
	No. of posters positioned by BHMK team	4	100	100	100	100	100
	Percentage of WIC foods marked with shelf labels by BHMK team	100%	100	100	100	100	100
	Percentage of posters remaining in place	≥75%	133.3	133.3	133.3	133.3	133.3
	Percentage of correctly positioned shelf labels remaining in place	≥80%	133.9	134.6	148.9	141.9	139.8
Product Placement							
	Percentage of WIC products moved by BHMK team per store	≥80%	125	125	125	125	125
	Percentage of products remaining at front of store and/or eye-level	≥80%	116.8	78.8	112.4	125	108.3
Grouping of Products							
	No. Displays assembled by BHMK team per store per treatment	1	100	100	100	100	100
	Percentage of time the display remained in place	≥80%	109.4	125	125	125	121.1
	Percentage of time the display contained only WIC products and required no maintenance by the BHMK team	≥80%	109.4	125	117.3	117.3	117.3

Abbreviations: BHMK (B’More Healthy Corner Stores for Moms and Kids); T (Treatment). ^a^ Percent reflects the high process evaluation standard. ^b^ Treatment 1 (one behavioral economic strategy), Treatment 2 (one behavioral economic strategy paired with store owner training), Treatment 3 (store owner training plus two behavioral economic strategies), Treatment 4 (all four behavioral economic strategies). ^c^ Overall average percentage of the high standard met for each process measure for whole intervention. ^d^ Additional videos may have been viewed online by store owners, but only in-person views are reported.

**Table 3:** Changes in stocking of WIC-eligible foods, total sales of WIC-eligible foods to all customers, and sales of WIC-eligible foods to WIC clients in intervention stores compared to control stores, following implementation of one BE strategy, for all food groups combined.

Changes ^1^	Store Owner Training (*n* = 2)	POP Promotion (*n* = 2)	Product Placement (*n* = 2)	Grouping of Products (*n* = 2)
	Mean (SE)	Mean (SE)	Mean (SE)	Mean (SE)
Stocking (No. of items)	251.0 (136.7)	40.0 (146.6)	168.0 (121.7)	−358.5 (287.1)
Total Sales (No. of items)	394.5 (319.1)	−149.0 (137.1)	−35.5 (54.1)	111.0 (66.0)
Sales to WIC Clients (No. of items)	60.0 (57.5)	−139.5 (110.3)	−76.5 (39.7)	−36.0 (112.8)

Abbreviations: SE (robust standard error), BE (behavioral economic), POP (point of purchase), WIC (Special Supplemental Nutrition Program for Women, Infants, and Children), No. (number). ^1^ Changes were calculated by subtracting values of the outcome immediately following Treatment 1 from baseline values and compared to the changes observed in control stores (*n* = 2, reference).

**Table 4:** Qualitative changes in the stocking of WIC foods, total unit sales of WIC foods to all customers, and unit sales of WIC foods to WIC clients in intervention stores compared to control stores, following implementation of one BE strategy, for different food groups.

Changes in the Stocking of WIC-eligible Foods by Food Group and BE Strategy ^1,2^					
WIC Food Groups	Store Owner Training *n* = 2	POP Promotion *n* = 2	Product Placement *n* = 2	Grouping of Products *n* = 2	Control *n* = 2
Infant Foods	+	+	+	+	-
Fruits and Vegetables	+	-	-	-	-
Dairy	+	-	+	-	+
Protein	+	+	+	-	+
Grains	+	+	-	-	-
Juice	+	-	-	-	-
Cereal	+	+	+	-	-
**Changes in the Total Unit Sales of WIC-eligible Foods to All Customers by Food Group and BE Strategy ^1,2^**					
Infant Foods	+	-	+	+	+
Fruits and Vegetables	+	-	-	+	-
Dairy	+	+	+	-	-
Protein	+	-	-	+	+
Grains	+	+	-	+	-
Juice	+	-	-	-	+
Cereal	+	-	-	+	+
**Changes in the Unit Sales of WIC-eligible Foods to WIC Clients by Food Group and BE Strategy ^1,2^**					
Infant Foods	-	-	-	+	+
Fruits and Vegetables	+	-	-	-	-
Dairy	+	+	+	-	+
Protein	+	-	-	+	-
Grains	+	+	-	-	-
Juice	+	-	-	-	-
Cereal	+	+	-	+	-

Abbreviations: BE (behavioral economic), POP (point of purchase), WIC (Special Supplemental Nutrition Program for Women, Infants, and Children). ^1^ Changes were calculated by subtracting values of the outcome immediately after Treatment 1 from baseline values. ^2^ Change ≥ 0 were coded as positive (+) and change < 0 as negative (-).

**Table 5:** Changes in the stocking of WIC-eligible foods, total unit sales of WIC-eligible foods to all customers, and unit sales of WIC-eligible foods to WIC clients in intervention stores following implementation of two, three, and four BE strategies, all food groups combined.

Changes ^1^	Number of BE Strategies Implemented			
	One *n* = 8	Two *n* = 8	Three *n* = 8	Four *n* = 8
	Reference	Mean (SE)	Mean (SE)	Mean (SE)
Stocking (No. of items)		10.5 (178.8)	−91.6 (149.0)	6.3 (94.1)
Total Sales (No. of items)		52.1 (123.1)	−69.9 (111.1)	−89.9 (96.6)
Sales to WIC Clients (No. of items)		78.1 (48.9)	26.9 (38.7)	27.6 (30.1)

Abbreviations: SE (robust standard error), BE (behavioral economic), WIC (Special Supplemental Nutrition Program for Women, Infants, and Children), No. (number). ^1^ Changes were calculated by subtracting values of the outcome immediately after each treatment from values before the treatment. The number of BE strategies implemented was treated as an ordinal variable (1 BE coded = 0; 2 BE coded = 1; 3 BE coded = 2; 4 BE coded = 3). Reference = change after treatment 1 (one BE strategy).

## Supplementary Materials

The following are available online at http://www.mdpi.com/1660-4601/16/1/64/s1, Figure S1: Sample BHMK posters by WIC-eligible developmental age group and food, Figure S2: Sample BHMK shelf label for fresh bananas, Figure S3: BHMK point of purchase promotion, Figure S4: Changes in average WIC redemption in USD by number of BE strategies implemented, during eWIC implementation, for BHMK intervention and control stores, Table S1: Food groups and individual food products classified according to WIC food products, Table S2: Changes in the stocking of WIC-eligiblefoods, total unit sales of WIC-eligiblefoods to all customers, and unit sales of WIC-eligiblefoods to WIC clients in intervention stores following implementation of two, three, and four BE strategies, by WIC food group.
