# Supplementary material for: B’more Healthy Corner Stores for Moms and Kids: Identifying Optimal Behavioral Economic Strategies to Increase WIC Redemptions in Small Urban Corner Stores
Source: Int J Environ Res Public Health. 2018 Dec 27;16(1):64. doi: 10.3390/ijerph16010064 (PMC6339089; doi:10.3390/ijerph16010064)
Supplement: Supplementary file 1 [file ijerph-16-00064-s001.pdf]

Supplemental Table S1. BHMK food groups and individual food products classified according to WIC-eligible food products.

Food Group	Individual
Infant	Formula, infant cereal, infant vegetables, infant fruit, infant mixed fruit and vegetables, infant meat.
Fruit & Vegetables	Fresh, frozen, and canned fruit varieties; Fresh, frozen, and canned vegetable varieties.
Dairy	Milk, cheese, yogurt, tofu, soy-based beverages.
Protein	Eggs, canned fish, dry beans, canned beans, dry peas, canned peas, dry lentils, canned lentils, peanut butter.
Grain	Whole wheat bread, whole wheat rolls, soft whole wheat tortillas, soft corn tortillas, dry brown rice.
Juice	100% juice frozen concentrate, 100% juice 64 oz.
Cereal	Cold breakfast cereals, oatmeal, cream of wheat, grits.

Abbreviations: BHMK (B'More Healthy Corner Stores for Moms and Kids), WIC (Special Supplemental Nutrition Program for Women, Infants, and Children).

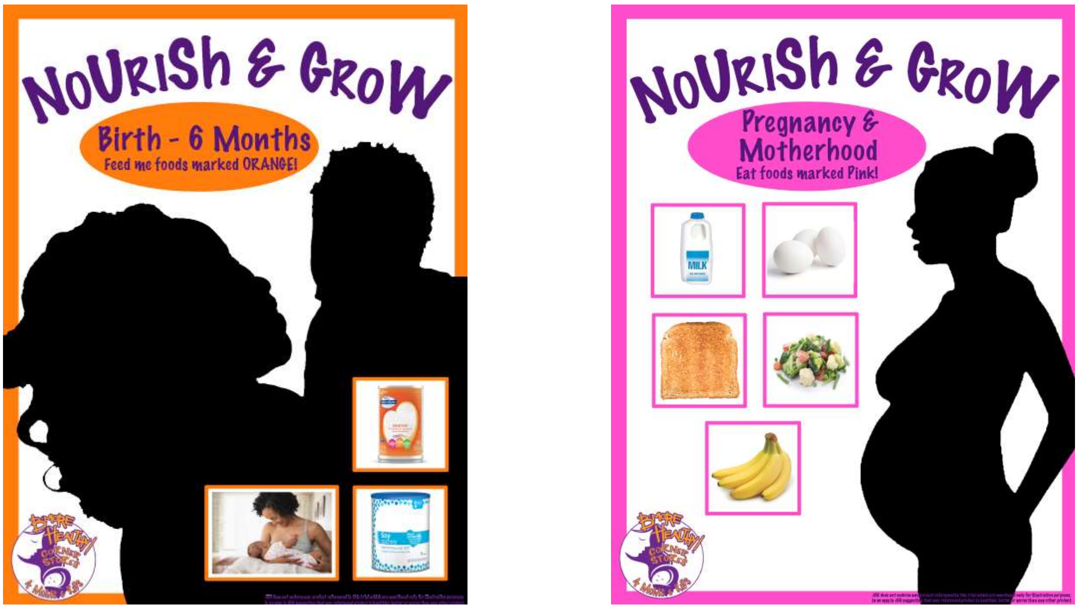

(**a**) (**b**)

Supplemental Figure S1. Sample BHMK posters by WIC-eligible developmental age group and food: (a) Orange was specific for children birth to six months; (b) Pink was specific for expecting mothers

Abbreviations: BHMK (B'More Healthy Corner Stores for Moms and Kids), WIC (Special Supplemental Nutrition Program for Women, Infants, and Children).

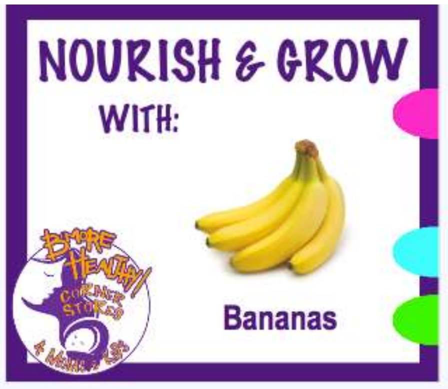

Supplemental Figure S2. Sample BHMK shelf label for fresh bananas. Pink, blue, and green thumb prints on the right side are indicative of bananas being a WIC-eligible food for expecting mothers, children six to twelve months of age, and children one to five years of age, respectively.

Abbreviations: BHMK (B'More Healthy Corner Stores for Moms and Kids).

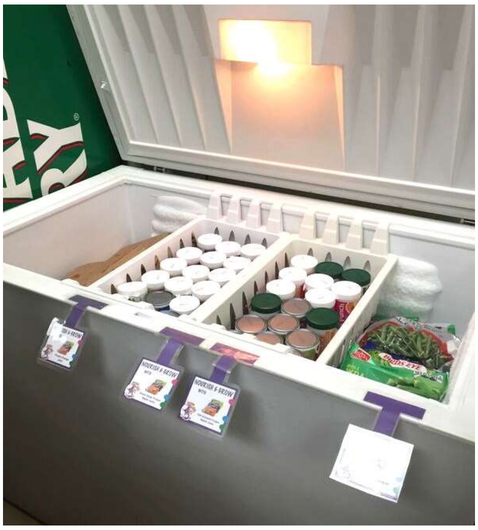

(**a**)

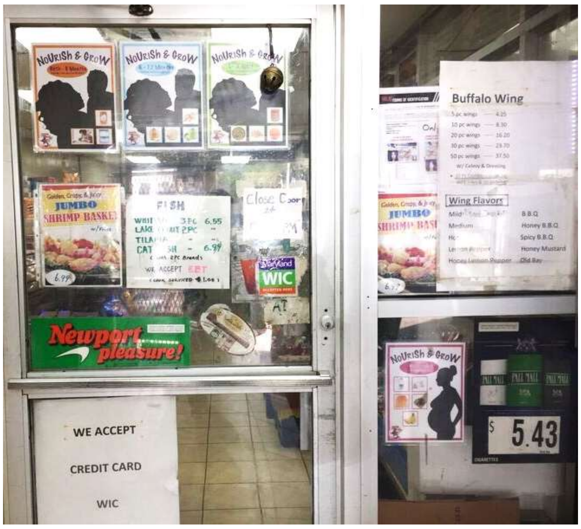

(**b**)

Supplemental Figure S3. BHMK POP promotion. (a) shelf labels on an unmarked freezer and (b) posters in a corner store entryway.

Abbreviations: BHMK (B'More Healthy Corner Stores for Moms and Kids), POP (point of purchase).

Supplemental Table S2. Changes in the stocking of WIC-eligible foods, total unit sales of WIC- eligible foods to all customers, and unit sales of WIC-eligible foods to WIC clients in intervention stores following implementation of two, three, and four BE strategies, by WIC food group.[1]

		Stocking (# of items)	Total Sales (# of items)	Sales to WIC clients (# of items)
	Infant food group	Mean change (SE)	Mean change (SE)	Mean change (SE)
Number of BE	1 treatment (reference)			
	2 treatments	-90.4 (41.5)	38.6 (17.0)	45.9 (12.4)*
	3 treatments	-78.4 (30.6)*	18.1 (28.1)	31.5 (25.3)
	4 treatments	-58.6 (29.6)	34.0 (19.4)	39.0 (19.8)
	Fruit and vegetables food group	Mean change (SE)	Mean change (SE)	Mean change (SE)
Number of BE	1 treatment (reference)			
	2 treatments	110.1 (96.6)	-4.0 (98.0)	29.5 (38.7)
	3 treatments	-7.1 (93.3)	-90.8 (89.4)	1.3 (27.9)
	4 treatments	71.8 (43.1)	-88.6 (64.0)	12.3 (24.7)
	Dairy food group	Mean change (SE)	Mean change (SE)	Mean change (SE)
Number of BE	1 treatment (reference)			
	2 treatments	14.8 (17.8)	4.3 (16.2)	-5.4 (9.9)
	3 treatments	3.9 (15.5)	0.1 (8.7)	-9.5 (6.4)
	4 treatments	3.0 (15.0)	-15.8 (15.1)	-15.5 (10.8)
	Protein food group	Mean change (SE)	Mean change (SE)	Mean change (SE)
Number of BE	1 treatment (reference)			
	2 treatments	-40.4 (22.3)	5.0 (7.9)	1.5 (3.7)
	3 treatments	-31.5 (20.8)	-7 (8.2)	-3.9 (4.9)
	4 treatments	-25.3 (11.3)	-16.6 (11.2)	-3.8 (6.7)
	Grains food group	Mean change (SE)	Mean change (SE)	Mean change (SE)
Number of BE	1 treatment (reference)			
	2 treatments	12.4 (13.1)	1.6 (7.1)	2.5 (4.7)
	3 treatments	13.8 (14.3)	0.6 (5.4)	2.4 (4.1)
	4 treatments	14.6 (19.2)	-1.4 (5.2)	0.8 (3.5)
	Juice food group	Mean change (SE)	Mean change (SE)	Mean change (SE)
Number of BE	1 treatment (reference)			
	2 treatments	10.5 (9.0)	6.6 (6.5)	3.5 (4.2)
	3 treatments	12.6 (11.6)	8.9 (5.8)	3.0 (3.4)
	4 treatments	5.1 (5.0)	3.3 (6.4)	-1.4(5.0)
	Cereal food group	Mean change (SE)	Mean change (SE)	Mean change (SE)
Number of BE	1 treatment (reference)			
	2 treatments	-6.5 (7.6)	0.0 (4.1)	0.6 (3.8)
	3 treatments	-4.9 (5.1)	0.1 (7.9)	2.1 (4.6)
	4 treatments	-4.4 (8.8)	-4.8 (5.8)	-3.8 (5.1)

Abbreviations: SE (robust standard error), BE (behavioral economic), WIC (Special Supplemental Nutrition Program for Women, Infants, and Children). [1]Changes were calculated by subtracting values of the outcome immediately after each treatment from values before the treatment. The number of BE strategies implemented was treated as an ordinal variable (1 BE coded=0; 2 BE coded=1; 3 BE coded=2; 4 BE coded=3). Reference = change after treatment 1 (one BE strategy). *p<0.05

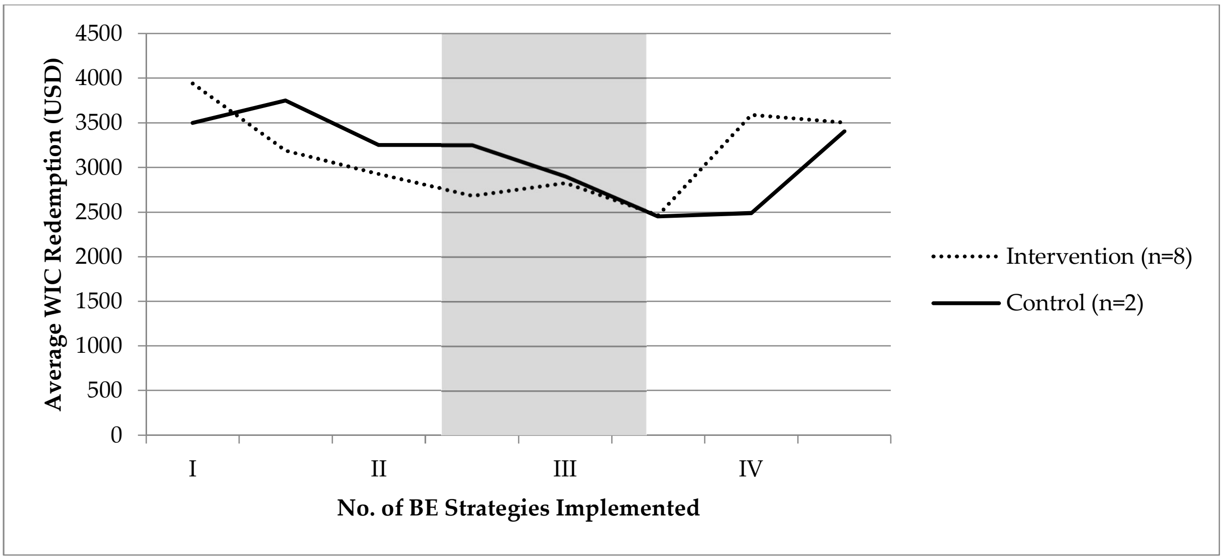

Supplemental Figure S4. Changes in average WIC redemption in USD by number of BE strategies implemented, during eWIC implementation, for BHMK intervention and control stores.

Abbreviations: BE (behavioral economic), WIC (Special Supplemental Nutrition Program for Women, Infants, and Children), BHMK (B'More Healthy Corner Stores for Moms and Kids), No. (number). The period during which eWIC was implemented is denoted by the gray box.
